# Evolution of pre- and post-operative balance characteristics in patients undergoing anterior cruciate ligament reconstruction: implications for rehabilitation

**DOI:** 10.3389/fbioe.2026.1861366

**Published:** 2026-06-25

**Authors:** Xiaoyang Xu, Yixin Zhang, Wanxue Wang, Heming Xu, Haipeng Liu, Peng Chen, Yan Xiao, Qinge Guo, Pengfei Yan, Haitao Fu, Chao Qi

**Affiliations:** Department of Sports Medicine, The Affiliated Hospital of Qingdao University, Qingdao, China

**Keywords:** anterior cruciate ligament reconstruction, gait analysis, limb symmetry, plantar pressure, return to sport, static and dynamic balance

## Abstract

**Background:**

Anterior cruciate ligament (ACL) injuries frequently necessitate anterior cruciate ligament reconstruction (ACLR), yet balance deficits persist during preoperative and postoperative periods. However, comprehensive longitudinal comparative studies remain scarce. This study utilized a high-resolution plantar pressure system to thoroughly assess these balance characteristics, aiming to optimize clinical rehabilitation.

**Methods:**

Fifty participants [25 in the ACLR group, 25 in the normal control (NC) group] were assessed using the SMARTX-MFS plantar pressure system. The ACLR group was assessed preoperatively, 2 months postoperatively, and 6 months postoperatively. The static parameters included the pressure proportion of the affected side (P_A_), the center of pressure (COP) 95% confidence ellipse area (S_COP_) and the COP trajectory length (L_COP_). The dynamic parameters included COP medial-lateral offset length (L_COP-ML_), COP anterior-posterior offset length (L_COP-AP_), gait line length (L_G_), single-leg support line length (L_S_), maximum moving speed of COP (V_MAX_), proportion of swing period (P_SW_), support period (P_S_), weight-bearing response period (P_WB_), single-leg support period (P_SL_), pre-swing period (P_SP_), and the maximum pressure in the forefoot (P_MAX-F_), arch (P_MAX-A_) and heel (P_MAX-H_). Inter-group and intra-group comparisons were conducted.

**Results:**

Preoperatively: The ACLR group exhibited impairments in both static and dynamic balance, alongside profound bilateral asymmetry. Two Months Postoperatively: Only L_COP-AP_ showed significant improvement (P > 0.05). Six Months Postoperatively: Static balance and most dynamic parameters showed no statistically significant differences compared to the NC group, except for increased L_COP-ML_ (P < 0.05) and decreased L_G_ and L_S_ of the affected side (P < 0.05). Within the ACLR group, the affected side still exhibited extremely significant decreased L_G_, L_S_, and P_MAX-F_ (P < 0.001), while P_MAX-A_ and P_MAX-H_ decreased significantly (P < 0.05).

**Conclusion:**

The ACLR group showed significant balance deficits at pre-operation. Only the L_COP-AP_ showed significant improvement at 2 months post-operation. The deficits basically recovered at 6 months post-operation. However, the asymmetry of COP trajectory and plantar pressure still existed. The study provided a basis for clinical rehabilitation assessment.

## 1 Introduction

Anterior cruciate ligament (ACL) injuries profoundly disrupt proprioceptive feedback and neuromuscular control, precipitating significant static and dynamic balance deficits (Kim et al., 2017; Pietrosimone et al., 2022; Huang et al., 2017). While anterior cruciate ligament reconstruction (ACLR) restores mechanical joint stability, substantial sensorimotor impairments frequently persist throughout the rehabilitation continuum (Pietrosimone et al., 2022; Hoch et al., 2017; Wang et al., 2023; Fleming et al., 2022; Moiroux-Sahraoui et al., 2025). Research indicates that although static balance typically normalizes within several months postoperatively, deficits in dynamic balance and multi-planar postural control often endure (Lennon et al., 2025; Erhart‐Hledik et al., 2018; Brophy et al., 2022; Shimizu et al., 2019; Lu et al., 2026). Notably, recent evidence demonstrates that dynamic kinetic abnormalities may persist long after isokinetic muscle strength has ostensibly recovered, thereby rendering conventional functional assessments insufficient when utilized in isolation (Moiroux-Sahraoui et al., 2025; Matov et al., 2025; Mittlmeier et al., 1999). These enduring biomechanical impairments generate abnormal kinematic and kinetic loading patterns in the lower extremities, thereby elevating the long-term risk of graft failure and posttraumatic osteoarthritis (PTOA) (Erhart‐Hledik et al., 2018; Shimizu et al., 2019; Lu et al., 2026; Webster and Hewett, 2022). Consequently, high-resolution plantar pressure systems have emerged as critical instruments for functional monitoring during orthopedic rehabilitation (Huang et al., 2017; Matov et al., 2025; Mittlmeier et al., 1999; Webster and Hewett, 2022; Çeti̇N et al., 2017; Gimunová et al., 2021). By quantitatively evaluating center of pressure (COP) trajectories, automatically delineating gait cycles, and mapping regional foot kinetics, these systems offer a precise, objective measure of both static postural stability and dynamic knee function (Huang et al., 2017; Matov et al., 2025; Webster and Hewett, 2022; Çeti̇N et al., 2017; Liu et al., 2026). However, comprehensive studies systematically evaluating the longitudinal evolution of these combined static and dynamic balance indices at various stages post-ACLR remain limited. To address this gap, our study employed the SMARTX-MFS plantar pressure system to prospectively examine changes in balance characteristics in the same cohort of patients at three distinct intervals: pre-operation, 2 months post-operation, and 6 months post-operation. The primary objective was to establish biomechanical reference data to optimize phase-specific clinical rehabilitation and to facilitate the continuous monitoring of functional changes.

## 2 Materials and methods

### 2.1 Participants

The recruitment period of this study was from 15 March 2024 to 15 June 2024. Participants who met the inclusion and exclusion criteria were selected and provided written informed consent. This study, approved by The Affiliated Hospital of Qingdao University (NO: QYFY WZLL 30167), involved 25 patients (14 males, 11 females) who underwent arthroscopic ACLR using autologous hamstring tendon for ACL injury and received routine rehabilitation training from the Department of Sports Medicine of The Affiliated Hospital of Qingdao University as the ACLR group, and 25 healthy volunteers (13 males, 12 females) without lower limb injury served as the normal control (NC) group. In the ACLR group, the mean interval from ACL injury to ACLR was 53.92 ± 19.95 ( $\bar{x}\pm s$ ) days. The two groups were comparable, exhibiting no statistically significant differences in age, body mass index (BMI) and gender distribution (P > 0.05) (Table 1). To ensure the reproducibility of the measurement data, we randomly sampled 25 healthy volunteers (12 males, 13 females) without lower limb injury to compare the intraclass correlation coefficient (ICC). Informed consent was obtained from all participants after a thorough explanation of the experimental requirements. The research was conducted in accordance with the guidelines outlined in the Declaration of Helsinki. All 50 cases in the ACLR group and the NC group were involved in result analysis. To verify the adequacy of the sample size, a *post hoc* power analysis was conducted using G*Power software (version 3.1.9.7). To detect a large effect size 0.8 with the α level of 0.05 and 0.8 power, the required total sample size was calculated to be 52 (26 per group) for the two independent samples t-test, and 15 for the paired samples t-test. Our actual enrolled sample of 50 participants (25 per group) closely approximates the requirement for between-group comparisons and meets the minimum sample size needed to detect large bilateral asymmetries within the ACLR group.

**TABLE 1:** Comparison of basic information of subjects in the two groups.

Variables		ACLR group (n = 25)	NC group (n = 25)	X^2^ or t-value	P-value
Age ( $\bar{x}\pm s,$ years)		30.00 ± 6.62	30.20 ± 8.10	−0.096	0.924
BMI ( $\bar{x}\pm s,$ kg/m^2^)		22.78 ± 1.91	25.06 ± 3.16	0.977	0.354
Gender	Male	14	13	0.081	0.777
	Female	11	12		

There was no significant difference in age, BMI, and gender distribution between the two groups (P > 0.05), which was comparable.

Inclusion criteria for ACLR group: (1) Age between 18 and 45 years (2) Initial unilateral ACL injury without other joint ligament damage. (3) Arthroscopic ACLR using autologous hamstring tendon graft. (4) Independent walking ability without assistive devices for ≥10 min at preoperative, 2-month postoperative, and 6-month postoperative assessments. (5) The time interval from ACL injury to ACLR is between 3 and 12 weeks (6) No history of musculoskeletal or neurological disorders affecting postural balance. (7) Informed consent after understanding study objectives and risks.

Exclusion criteria for ACLR group: (1) Persistent pain or surgical history of hip, knee, ankle, or foot in the past 6 months. (2) Concomitant grade III–IV cartilage damage, meniscectomy >1/3, or meniscal repair. (3) Postoperative complications (infection, arthrofibrosis, deep vein thrombosis, etc.). (4) Non-compliance or inability to complete the experiment. (5) Allergy to experimental materials.

Inclusion criteria for the NC group: (1) Age, gender, and BMI matched to the ACLR group. (2) No history of lower extremity fractures, ligament injuries, or surgery. (3) Absence of neuromusculoskeletal disorders affecting gait. (4) Normal bilateral knee function.

### 2.2 Procedure

#### 2.2.1 Pre-test preparation

The experiment took place in a noise-controlled and well-lit room to minimize auditory and vision interference. To limit footwear-related variability, all participants wore cotton socks of identical thickness during measurements. One day before the test, participants avoided high-intensity activities and then walked freely for 5 min in the standardized socks to establish a natural gait. For each participant, the SMARTX-MFS system was calibrated twice as per the manual to ensure the equipment worked properly. Important participant details—name, gender, height, weight, and date—were recorded prior to the experiment.

#### 2.2.2 Equipment

**FIGURE 1:**
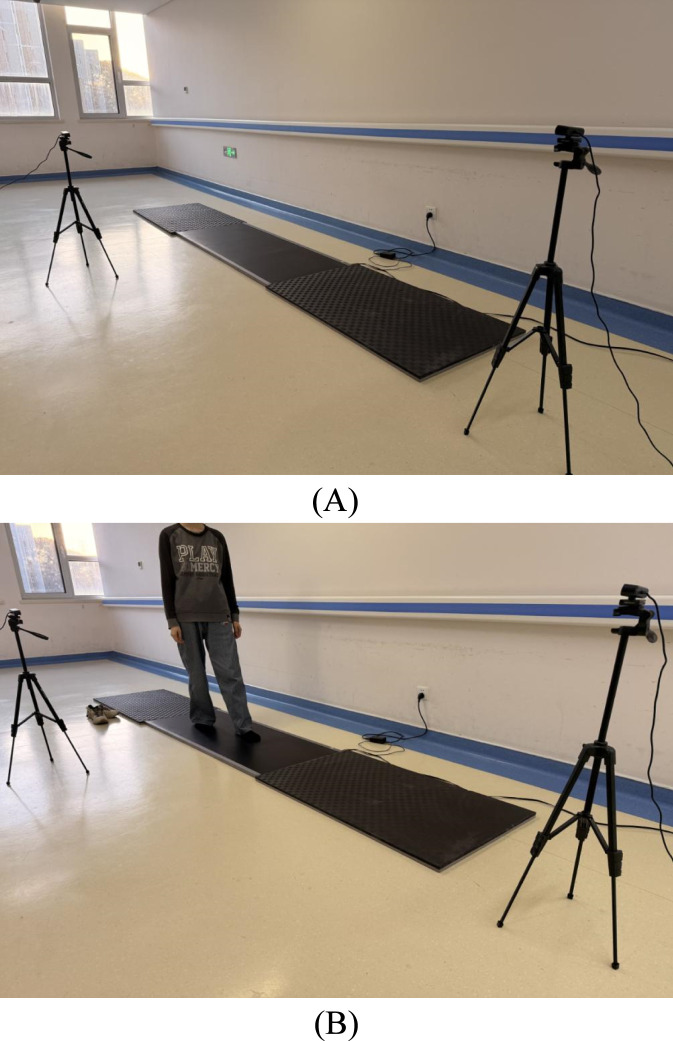
Plantar pressure plate (SMARTX-MFS) and measurement process. **(A)** Plantar pressure plate (SMARTX-MFS). **(B)** Measurement process.

In this experiment, the SMARTX-MFS plantar pressure and gait assessment system (version 1.0, produced by Qingdao Intelligent Sense Technology Co. Ltd., Patent No.: CN213309713U) was used to collect data. The system features a high-precision pressure sensing platform [158.0 cm × 66.5 cm × 2.62 cm (L × W × H)] incorporating capacitive pressure sensors at a density of 1.4 sensors/cm^2^. The sampling frequency is 100 Hz. The accompanying SMARTX-MFS software enables real-time calculation of COP trajectory coordinates from plantar pressure distribution data. The measurement system is connected to a PC via USB. A rubber extension cushion [105.0 cm × 66.5 cm × 2.62 cm (L × W × H)] was securely attached to each side of the plantar pressure plate. These cushions served as buffer zones, ensuring participants maintain a stable gait within the data collection area. Figure 1 shows the device. To assess the stability of data measured by the device, we conducted an ICC analysis involving 25 previously selected healthy volunteers. First, three randomly selected operators each conducted a single test on every participant. Next, the same operator performed two tests on each participant, with a 1-day interval between tests. Following these procedures, ICC analysis was conducted separately for each scenario. For inter-operator consistency, the absolute-consistency single-measurement ICC from a two-way random-effects model was utilized. For test-retest consistency, the absolute-consistency single-measurement ICC from a two-way mixed-effects model was employed. Additionally, the 95% confidence intervals (95% CIs) were reported for both analyses. The specific test procedures were all carried out as described in Section 2.2.3. An ICC greater than 0.75 was the threshold for determining good consistency. The ICC values of the inter-operator consistency analysis ranged from 0.800 [95%CIs (0.656, 0.898)] to 0.922 [95%CIs (0.857, 0.962)]. The ICC values of the test-retest consistency analysis ranged from 0.798 [95%CIs (0.596, 0.906)] to 0.957 [95%CIs (0.905, 0.981)]. The results indicated that all indicators achieved an ICC above 0.75, demonstrating the device’s reliable stability in repeated measurements. Detailed ICC values and their 95%CIs are presented in Supplementary Tables S1, S2.

#### 2.2.3 Testing procedure

Participants in the ACLR group were tested 3 days before surgery, and again at two and 6 months post-operation. The NC group did not have specific test time requirements. Before the static test, participants stood on a plantar pressure plate with feet shoulder-width apart, arms relaxed, and eyes fixed on a visual marker for 10 s to acclimate. Then the system recorded balance metrics during a 10-s natural standing period to complete the static balance test. Following this, the dynamic balance test was conducted. Participants walked back and forth on a test runway for 2 min at a self-selected natural speed to acclimate. The use of a self-selected natural speed aimed to truly reflect the dynamic balance defects of participants in daily walking, avoiding the artificial alteration of dynamic balance metrics that can occur when forcibly setting a fixed speed (such as using a forced-pace treadmill or an auditory metronome). After finishing the adaptive training, the participants commenced walking from one side of the extension cushion. They adopted a self-selected natural speed, stepped onto the plantar pressure plate, walked to the endpoint on the opposite extension cushion, and then returned to the starting point. This back-and-forth walking continued for 60 s to ensure the collection of at least 15 complete and stable gait cycles. During this time, the system gathered dynamic balance data. The above test process was conducted three times, with a 5-min interval between repetitions. If data collection was incomplete, participants repeated the process.

#### 2.2.4 Data processing

Due to the visibility of surgical scars in the ACLR group, on-site operators could not be blinded to the participants’ group assignments or limb conditions. However, after data collection for all groups and time points, the dedicated data collectors exported the data in PDF format and organized it into XLS format. The data collectors first calculated the average of the data from three tests for each participant. Then, to streamline further analysis, they averaged the data for the bilateral lower limbs in the NC group. After that, they encoded the groups and limb states with letters and numbers before submitting the data to statistical analysts. This process aimed to reduce subjective bias in the statistical analysis. In the subsequent illustration of bilateral lower limb results within the ACLR group, we define the “affected side” as the leg that underwent ACLR. Conversely, the “unaffected side” refers to the healthy contralateral leg that remained uninjured.

**FIGURE 2:**
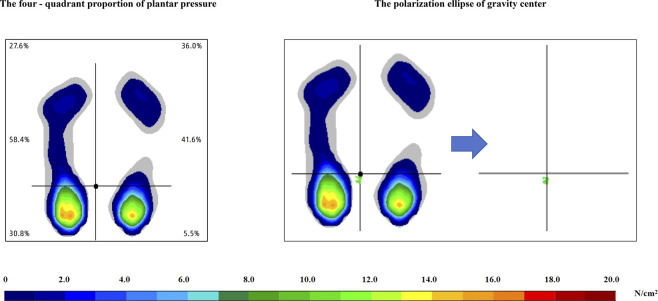
The data related to static balance.

Static balance metrics: pressure proportion of affected side (P_A_), the COP 95% confidence ellipse area (S_COP_), the COP trajectory length (L_COP_) (Figure 2). In the NC group, both lower limbs were healthy. To facilitate subsequent statistical analysis and interpretation of results, the P_A_ for the NC group was calculated using the average of the pressure proportions from both lower limbs, as previously mentioned. S_COP_ indicates the area of COP sway during static standing, while L_COP_ denotes the total length of the COP movement trajectory. Larger values for these parameters indicate greater variation in the COP of the participants during static standing, which also means a greater challenge to balance control.

**FIGURE 3:**
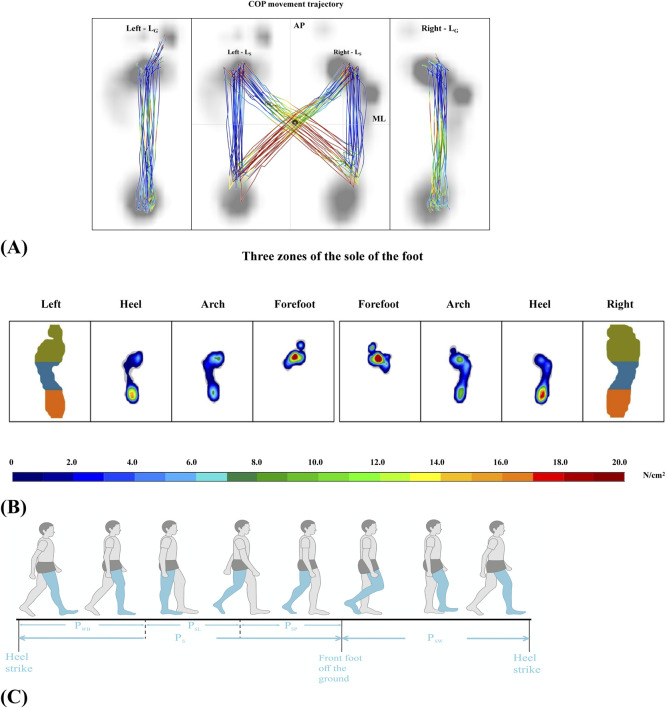
The data related to dynamic balance. **(A)** The COP movement trajectory and related data. **(B)** The maximum pressure of the three partitions on the sole. **(C)** Schematic diagram of gait cycle (taking the right lower limb as an example).

Dynamic balance metrics: COP medial-lateral offset length (L_COP-ML_), COP anterior-posterior offset length (L_COP-AP_), gait line length (L_G_), single-leg support line length (L_S_), maximum moving speed of COP (V_MAX_), proportion of swing period (P_SW_), support period (P_S_), weight-bearing response period (P_WB_), single-leg support period (P_SL_), pre-swing period (P_SP_), and the maximum pressure in the forefoot (P_MAX-F_), arch (P_MAX-A_), heel (P_MAX-H_) (Figure 3). The COP trajectory of one foot during walking is termed the gait line, while during the single leg support phase, it is referred to as the single-leg support line. During dynamic walking, the COP shifts continuously between the soles of both feet. This movement creates a trajectory known as the butterfly diagram. V_MAX_ is defined as the maximum velocity of the COP movement during walking. The central region’s position on the butterfly diagram’s coordinate axes indicates the COP offset in both the medial-lateral and anterior-posterior directions during movement. Plantar pressure distribution was analyzed using the system’s built-in anatomical segmentation algorithm, which automatically divided the foot into three regions (forefoot, arch, and heel) and calculated maximum pressure for each region. Forefoot pressure indicates propulsion efficiency during push-off. Arch pressure signifies arch support and shock absorption. Heel pressure demonstrates stability during initial contact (Liu et al., 2025; Liu et al., 2024; Arzehgar et al., 2025). The system software automatically identified gait cycles using a pressure threshold method. The gait cycle consists of the support period and the swing period. The support period is further subdivided into the weight-bearing response period, single-leg support period, and pre-swing period.

### 2.3 Statistical analysis

Data analysis was performed using SPSS (version 29.0; IBM) and Excel 2016 (Microsoft, Redmond, Washington, United States). The original bar chart was generated with GraphPad Prism software (version 10.6.1), while the forest plot was generated using R software (version 4.5.1). Continuous variables were expressed as mean ± standard deviation ( $\bar{x}\pm s$ ). Normality was assessed using the Shapiro-Wilk test. For normally distributed data, the independent samples t-tests were used for inter-group comparisons, and the paired samples t-tests were used for intra-group comparisons. If the normal distribution was not satisfied, M (P25, P75) was used, Mann-Whitney U test was used between groups, and Wilcoxon signed rank test was used within groups. Prior to inferential statistical analysis, the assumption of normality for all continuous variables was rigorously assessed using the Shapiro-Wilk test, which confirmed that all data met the normality assumption (P > 0.05). Under this baseline of normality, the Grubbs criterion was further applied to verify the absence of potential statistical outliers; the analysis indicated that no significant outliers were identified across any variables, and no observations were excluded. Consequently, parametric tests were utilized. The significance level (α) was set at 0.05. A P-value of less than 0.05 indicates that the difference is statistically significant, while a P-value of less than 0.001 indicates an extremely significant statistical difference. Given the multidimensional and correlated nature of the biomechanical parameters assessed, this study was designed as an exploratory data analysis. Consequently, to avoid an overly conservative inflation of Type II errors that could mask clinically relevant rehabilitation signals, adjustments for multiple comparisons (e.g., Bonferroni correction) were not applied to the family-wise error rate (Rothman, 1990; Kimmelman et al., 2014; Ditroilo et al., 2025). To ensure full statistical transparency and mitigate the risk of misinterpretation caused by Type I error inflation, we reported the P-values alongside effect size estimates (Cohen’s d) and 95% CIs. This dual reporting strategy allowed readers to better evaluate the clinical relevance of the findings. In our study, we took the absolute value of Cohen’s d for the comparison of the effect size and defined a Cohen’s d of less than 0.2 as extremely small effect size, 0.2 or higher as small, 0.5 or higher as medium, 0.8 or higher as large. In contrast, sports medicine and biomechanics research often observes Cohen’s d values exceeding 1.2 due to significant changes in kinematic and kinetic parameters in diseased states. We classified these instances as having an extremely large effect size.

## 3 Results

Detailed descriptive statistics $\left(\bar{x}\pm s\right)$ , t-values, P-values and Cohen’s d (with 95%CIs) can be found in Tables 2–10. To elucidate the data comparisons, we provided additional explanations using the magnitude of the effect sizes and Cohen’s d (95%CIs) for some indicators with significant differences at 6 months post-operation to facilitate the understanding and subsequent application by clinical readers.

**TABLE 2:** Comparison of static balance parameters between the ACLR group and the NC group at pre-operation ( $\bar{x}\pm s$ ).

Parameters	ACLR group (n = 25)	NC group (n = 25)	t-value	P-value	Cohen’s d 95% (CI)
P_A_ (%)	42.76 ± 2.09	49.81 ± 0.45	16.472	<0.001	4.66 [3.57, 5.73]
S_COP_ (mm^2^)	39.84 ± 9.56	10.76 ± 3.10	−16.742	<0.001	−4.09 [-5.07, −3.10]
L_COP_ (mm)	170.36 ± 18.70	104.60 ± 6.00	−14.474	<0.001	−4.74 [-5.82, −3.63]

**TABLE 3:** Comparison of dynamic balance parameters between the ACLR group and the NC group at pre-operation ( $\bar{x}\pm s$ ).

​	Parameters	ACLR group (n = 25)	NC group (n = 25)	t-value	P-value	Cohen’s d (95%CI)
Affected side	P_S_ (%)	60.77 ± 1.71	60.00 ± 0.52	−2.136	0.038	−0.62 [-1.19, −0.05]
	P_SW_ (%)	38.63 ± 1.18	39.80 ± 0.85	3.985	<0.001	0.42 [-0.14, 0.98]
	P_WB_ (%)	11.27 ± 1.21	9.67 ± 0.43	−6.223	<0.001	−1.73 [-2.37, −1.07]
	P_SL_ (%)	35.10 ± 1.05	40.40 ± 0.76	5.968	<0.001	5.57 [4.32, 6.81]
	P_SP_ (%)	11.25 ± 1.21	9.72 ± 0.43	−20.395	<0.001	−1.72 [-2.37, −1.06]
	L_G_ (mm)	147.62 ± 13.57	189.44 ± 9.11	12.873	<0.001	3.55 [2.65, 4.45]
	L_S_ (mm)	88.56 ± 9.33	125.58 ± 7.17	15.838	<0.001	4.38 [3.34, 5.40]
	P_MAX-F_ (N/cm^2^)	16.26 ± 2.04	22.62 ± 2.72	9.340	<0.001	2.62 [1.85, 3.37]
	P_MAX-A_ (N/cm^2^)	6.19 ± 0.71	9.61 ± 2.18	7.471	<0.001	2.12 [1.41, 2.81]
	P_MAX-H_ (N/cm^2^)	18.57 ± 0.95	26.23 ± 1.60	20.640	<0.001	5.79 [4.50, 7.06]
Unaffected side	P_S_ (%)	63.76 ± 1.86	60.00 ± 0.52	−9.711	<0.001	−2.75 [-3.52, −1.96]
	P_SW_ (%)	36.44 ± 1.39	39.80 ± 0.85	10.360	<0.001	2.90 [2.09, 3.69]
	P_WB_ (%)	12.92 ± 1.23	9.67 ± 0.43	−17.003	<0.001	−3.48 [-4.36, −2.59]
	P_SL_ (%)	38.45 ± 1.36	40.40 ± 0.76	7.292	<0.001	1.75 [1.09, 2.40]
	P_SP_ (%)	13.56 ± 1.04	9.72 ± 0.43	−6.280	<0.001	−4.83 [-5.94, −3.71]
	L_G_ (mm)	172.40 ± 9.61	189.44 ± 9.11	6.497	<0.001	1.80 [1.13, 2.45]
	L_S_ (mm)	104.33 ± 8.20	125.58 ± 7.17	9.833	<0.001	2.71 [1.93, 3.48]
	P_MAX-F_ (N/cm^2^)	17.67 ± 2.03	22.62 ± 2.72	7.292	<0.001	2.04 [1.35, 2.73]
	P_MAX-A_ (N/cm^2^)	6.96 ± 0.66	9.61 ± 2.18	5.829	<0.001	1.65 [1.00, 2.29]
	P_MAX-H_ (N/cm^2^)	20.22 ± 1.18	26.23 ± 1.60	15.139	<0.001	4.25 [3.23, 5.26]
L_COP-ML_ (mm)		16.13 ± 4.84	1.32 ± 0.98	−14.997	<0.001	−4.24 [-5.25, −3.22]
L_COP-AP_ (mm)		14.18 ± 2.93	3.18 ± 1.09	−17.614	<0.001	−4.88 [-6.00, −3.76]
V_MAX_ (m/s)		4.50 ± 0.50	2.89 ± 0.17	−15.284	<0.001	−4.34 [-5.36, −3.30]

**TABLE 4:** Comparison of dynamic balance parameters on the affected side and the unaffected side in the ACLR group at pre-operation (n = 25, $\bar{x}\pm s$ ).

Parameters	Affected side	Unaffected side	t-value	P-value	Cohen’s d (95%CI)
P_S_ (%)	60.77 ± 1.71	63.76 ± 1.86	−6.652	<0.001	−1.33 [-1.87, −0.78]
P_SW_ (%)	38.63 ± 1.18	36.44 ± 1.39	6.065	<0.001	1.37 [0.81, 1.91]
P_WB_ (%)	11.27 ± 1.21	12.92 ± 1.23	−6.109	<0.001	−1.22 [-1.74, −0.69]
P_SL_ (%)	35.10 ± 1.05	38.45 ± 1.36	−10.851	<0.001	−2.17 [-2.89, −1.44]
P_SP_ (%)	11.25 ± 1.21	13.56 ± 1.04	−9.388	<0.001	−1.88 [-2.53, −1.21]
L_G_ (mm)	147.62 ± 13.57	172.40 ± 9.61	−13.057	<0.001	−2.51 [-3.31, −1.70]
L_S_ (mm)	88.56 ± 9.33	104.33 ± 8.20	−14.448	<0.001	−2.78 [-3.64, −1.90]
P_MAX-F_ (N/cm^2^)	16.26 ± 2.04	17.67 ± 2.03	−5.823	<0.001	−1.17 [-1.67, −0.65]
P_MAX-A_ (N/cm^2^)	6.19 ± 0.71	6.96 ± 0.66	−6.900	<0.001	−1.38 [-1.92, −0.82]
P_MAX-H_ (N/cm^2^)	18.57 ± 0.95	20.22 ± 1.18	−10.028	<0.001	−2.01 [-2.69, −1.31]

**TABLE 5:** Comparison of static balance parameters between the ACLR group and the NC group at 2 months post-operation ( $\bar{x}\pm s$ ).

Parameters	ACLR group (n = 25)	NC group (n = 25)	t-value	P-value	Cohen’s d (95%CI)
P_A_ (%)	45.95 ± 1.68	49.81 ± 0.45	11.098	<0.001	3.14 [2.30, 3.97]
S_COP_ (mm^2^)	27.20 ± 7.33	10.76 ± 3.10	−10.320	<0.001	−2.92 [-3.72, −2.11]
L_COP_ (mm)	151.20 ± 11.64	104.60 ± 6.00	−17.796	<0.001	−5.03 [-6.17, −3.88]

**TABLE 6:** Comparison of dynamic balance parameters between the ACLR group and the NC group at 2 months post-operation ( $\bar{x}\pm s$ ).

​	Parameters	ACLR group (n = 25)	NC group (n = 25)	t-value	P-value	Cohen’s d (95%CI)
Affected side	P_S_ (%)	61.05 ± 1.44	60.00 ± 0.52	−3.405	<0.001	−0.98 [-1.56, −0.39]
	P_SW_ (%)	38.82 ± 1.57	39.80 ± 0.85	2.735	<0.001	0.78 [0.20, 1.36]
	P_WB_ (%)	10.96 ± 0.83	9.67 ± 0.43	−6.913	<0.001	−1.90 [-1.22, −2.57]
	P_SL_ (%)	36.29 ± 1.76	40.40 ± 0.76	10.755	<0.001	3.00 [2.18, 3.81]
	P_SP_ (%)	11.20 ± 1.03	9.72 ± 0.43	−6.655	<0.001	−1.92 [-2.59, −1.24]
	L_G_ (mm)	158.14 ± 12.15	189.44 ± 9.11	10.309	<0.001	2.90 [2.09, 3.70]
	L_S_ (mm)	95.33 ± 8.16	125.58 ± 7.17	13.921	<0.001	3.92 [2.96, 4.87]
	P_MAX-F_ (N/cm^2^)	18.81 ± 1.32	22.62 ± 2.72	6.295	<0.001	1.76 [1.10, 2.41]
	P_MAX-A_ (N/cm^2^)	7.40 ± 0.62	9.61 ± 2.18	4.872	<0.001	1.37 [0.75, 2.00]
	P_MAX-H_ (N/cm^2^)	20.70 ± 0.97	26.23 ± 1.60	14.803	<0.001	4.15 [3.15, 5.14]
Unaffected side	P_S_ (%)	62.42 ± 1.23	60.00 ± 0.52	−9.089	<0.001	−2.56 [-3.31, −1.80]
	P_SW_ (%)	37.47 ± 1.39	39.80 ± 0.85	7.143	<0.001	2.00 [1.31, 2.68]
	P_WB_ (%)	11.74 ± 1.07	9.67 ± 0.43	−8.956	<0.001	−2.49 [-3.23, −1.74]
	P_SL_ (%)	38.51 ± 1.34	40.40 ± 0.76	6.160	<0.001	1.71 [1.06, 2.36]
	P_SP_ (%)	12.40 ± 1.03	9.72 ± 0.43	−12.000	<0.001	−3.42 [-4.30, −2.54]
	L_G_ (mm)	173.00 ± 9.86	189.44 ± 9.11	6.127	<0.001	1.73 [1.07, 2.38]
	L_S_ (mm)	104.59 ± 6.77	125.58 ± 7.17	10.642	<0.001	3.00 [2.17, 3.80]
	P_MAX-F_ (N/cm^2^)	19.71 ± 1.12	22.62 ± 2.72	4.933	<0.001	1.37 [0.75, 1.99]
	P_MAX-A_ (N/cm^2^)	8.01 ± 0.49	9.61 ± 2.18	3.580	<0.001	1.00 [0.41, 1.59]
	P_MAX-H_ (N/cm^2^)	21.62 ± 0.84	26.23 ± 1.60	12.785	<0.001	3.59 [2.67, 4.48]
L_COP-ML_ (mm)		10.03 ± 1.93	1.32 ± 0.98	−20.116	<0.001	−5.69 [-6.95, −4.42]
L_COP-AP_ (mm)		3.28 ± 1.05	3.18 ± 1.09	0.315	0.754	−0.93 [-1.51, −0.34]
V_MAX_ (m/s)		3.57 ± 0.32	2.89 ± 0.17	−3.288	<0.001	−2.67 [-3.43, −1.89]

**TABLE 7:** Comparison of dynamic balance parameters on the affected side and the unaffected side in the ACLR group at 2 months post-operation (n = 25, $\bar{x}\pm s$ ).

Parameters	Affected side	Unaffected side	t-value	P-value	Cohen’s d (95%CI)
P_S_ (%)	61.05 ± 1.44	62.42 ± 1.23	−6.908	<0.001	−1.38 [-1.93, −0.82]
P_SW_ (%)	38.82 ± 1.57	37.47 ± 1.39	6.866	<0.001	1.37 [-0.82, 1.92]
P_WB_ (%)	10.96 ± 0.83	11.74 ± 1.07	−7.482	<0.001	−1.50 [-2.06, −0.91]
P_SL_ (%)	36.29 ± 1.76	38.51 ± 1.34	−8.345	<0.001	−1.67 [-2.27, −1.05]
P_SP_ (%)	11.20 ± 1.03	12.40 ± 1.03	−8.409	<0.001	−1.68 [-2.29, −1.06]
L_G_ (mm)	158.14 ± 12.15	173.00 ± 9.86	−7.883	<0.001	1.58 [-2.16, −0.98]
L_S_ (mm)	95.33 ± 8.16	104.59 ± 6.77	−8.321	<0.001	−1.66 [-2.27, −1.05]
P_MAX-F_ (N/cm^2^)	18.81 ± 1.32	19.71 ± 1.12	−7.944	<0.001	−1.59 [-2.18, −0.99]
P_MAX-A_ (N/cm^2^)	7.40 ± 0.62	8.01 ± 0.49	−6.813	<0.001	−1.36 [-1.90, −0.81]
P_MAX-H_ (N/cm^2^)	20.70 ± 0.97	21.62 ± 0.84	−8.698	<0.001	−1.74 [-2.36, −1.11]

**TABLE 8:** Comparison of static balance parameters between the ACLR group and the NC group at 6 months post-operation ( $\bar{x}\pm s$ ).

Parameters	ACLR group (n = 25)	NC group (n = 25)	t-value	P-value	Cohen’s d (95%CI)
P_A_ (%)	49.78 ± 0.42	49.81 ± 0.45	0.194	0.847	1.28 [0.67, 1.89]
S_COP_ (mm^2^)	10.96 ± 3.27	10.76 ± 3.10	−0.222	0.825	−1.84 [-2.50, −1.17]
L_COP_ (mm)	104.70 ± 5.45	104.60 ± 6.00	−0.049	0.961	−2.50 [-3.24, −1.75]

**TABLE 9:** Comparison of dynamic balance parameters between the ACLR group and the NC group at 6 months post-operation ( $\bar{x}\pm s$ ).

​	Parameters	ACLR group (n = 25)	NC group (n = 25)	t-value	P-value	Cohen’s d (95%CI)
Affected side	P_S_ (%)	60.28 ± 0.49	60.00 ± 0.52	−1.933	0.059	−0.73 [-1.30, −0.15]
	P_SW_ (%)	39.40 ± 0.69	39.80 ± 0.85	1.785	0.081	0.80 [0.22, 1.38]
	P_WB_ (%)	9.85 ± 0.33	9.67 ± 0.43	−1.817	0.076	−1.20 [-1.80, −0.59]
	P_SL_ (%)	39.98 ± 0.72	40.40 ± 0.76	2.006	0.051	0.92 [0.34, 1.50]
	P_SP_ (%)	9.94 ± 0.35	9.72 ± 0.43	−1.994	0.052	−1.27 [-1.87, −0.65]
	L_G_ (mm)	184.61 ± 7.42	189.44 ± 9.11	2.059	0.045	0.60 [0.03, 1.16]
	L_S_ (mm)	121.49 ± 5.02	125.58 ± 7.17	2.336	0.024	0.65 [0.08, 1.22]
	P_MAX-F_ (N/cm^2^)	21.92 ± 1.63	22.62 ± 2.72	1.104	0.275	0.30 [-0.25, 0.86]
	P_MAX-A_ (N/cm^2^)	9.38 ± 0.72	9.61 ± 2.18	0.489	0.627	0.12 [-0.44, 0.67]
	P_MAX-H_ (N/cm^2^)	25.88 ± 0.97	26.23 ± 1.60	0.952	0.346	0.27 [-0.29, 0.83]
Unaffected side	P_S_ (%)	60.23 ± 0.49	60.00 ± 0.52	−1.599	0.116	−1.09 [-1.68, −0.49]
	P_SW_ (%)	39.41 ± 0.68	39.80 ± 0.85	1.760	0.085	1.63 [0.98, 2.27]
	P_WB_ (%)	9.82 ± 0.29	9.67 ± 0.43	−1.629	0.110	−1.11 [-1.70, −0.51]
	P_SL_ (%)	40.03 ± 0.61	40.40 ± 0.76	1.907	0.062	0.69 [0.12, 1.26]
	P_SP_ (%)	9.90 ± 0.27	9.72 ± 0.43	−1.739	0.088	−1.33 [-1.94, −0.71]
	L_G_ (mm)	185.86 ± 7.69	189.44 ± 9.11	1.503	0.139	0.45 [-0.12, 1.01]
	L_S_ (mm)	122.50 ± 5.17	125.58 ± 7.17	1.738	0.089	0.48 [-0.09, 1.04]
	P_MAX-F_ (N/cm^2^)	22.40 ± 1.63	22.62 ± 2.72	0.347	0.730	0.09 [-0.46, 0.65]
	P_MAX-A_ (N/cm^2^)	9.56 ± 0.70	9.61 ± 2.18	0.105	0.917	0.01 [-0.55, 0.56]
	P_MAX-H_ (N/cm^2^)	26.25 ± 0.86	26.23 ± 1.60	−0.055	0.956	−0.01 [-0.56, 0.55]
L_COP-ML_ (mm)		1.83 ± 0.72	1.32 ± 0.98	−2.090	0.042	−1.59 [-2.22, −0.94]
L_COP-AP_ (mm)		3.48 ± 0.86	3.18 ± 1.09	−1.051	0.298	−0.30 [-0.85, 0.26]
V_MAX_ (m/s)		2.90 ± 0.16	2.89 ± 0.17	0.067	0.947	0.02 [-0.53, 0.58]

**TABLE 10:** Comparison of dynamic balance parameters on the affected side and the unaffected side in ACLR group at 6 months post-operation (n = 25, $\bar{x}\pm s$ ).

Parameters	Affected side	Unaffected side	t-value	P-value	Cohen’s d (95%CI)
P_S_ (%)	60.28 ± 0.49	60.23 ± 0.49	1.130	0.270	−1.00 [-1.47, −0.51]
P_SW_ (%)	39.40 ± 0.69	39.41 ± 0.68	−0.272	0.788	−1.55 [-0.96, 2.13]
P_WB_ (%)	9.85 ± 0.33	9.82 ± 0.29	1.098	0.283	−0.45 [-0.86, −0.03]
P_SL_ (%)	39.98 ± 0.72	40.03 ± 0.61	−0.583	0.565	−0.60 [-1.03, −0.17]
P_SP_ (%)	9.94 ± 0.35	9.90 ± 0.27	1.000	0.327	−0.47 [-0.88, −0.05]
L_G_ (mm)	184.61 ± 7.42	185.86 ± 7.69	−7.944	<0.001	−1.59 [-2.18, −0.99]
L_S_ (mm)	121.49 ± 5.02	122.50 ± 5.17	−3.874	<0.001	−0.78 [-1.22, −0.32]
P_MAX-F_ (N/cm^2^)	21.92 ± 1.63	22.40 ± 1.63	−6.766	<0.001	−1.35 [-1.89, −0.80]
P_MAX-A_ (N/cm^2^)	9.38 ± 0.72	9.56 ± 0.70	−2.827	0.009	−0.57 [-0.98, −0.14]
P_MAX-H_ (N/cm^2^)	25.88 ± 0.97	26.25 ± 0.86	−3.525	0.002	−0.71 [-1.14, −0.26]

### 3.1 Comparison of the ACLR group and the NC group at pre-operation

**FIGURE 4:**
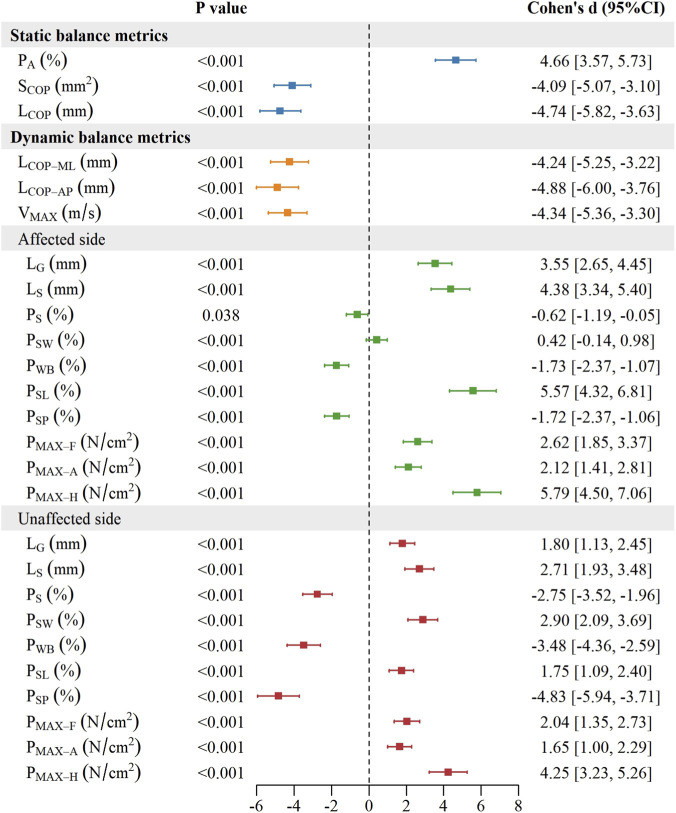
Comparison of balance parameters between the ACLR group and the NC group at pre-operation. The forest plot shows the P-value, Cohen’s d, 95% CI of the balance parameters and direction indicates the ACLR group vs. the NC group.

**FIGURE 5:**
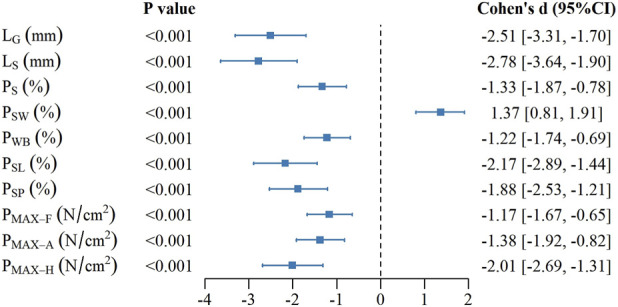
Comparison of balance parameters within the ACLR group at pre-operation. The forest plot shows the P-value, Cohen’s d, 95% CI of the balance parameters and direction indicates affected side vs. unaffected side within the ACLR group.

Compared with the NC group, the ACLR group exhibited the decreased P_A_ and the increased S_COP_ and L_COP_. The differences were extremely significant (P < 0.001) (Table 2). L_COP-ML_, L_COP-AP_ and V_MAX_ increased extremely significantly in the ACLR group compared with the NC group during walking (P < 0.001). P_S_ of the affected side increased significantly (P < 0.05), which was extremely significant on the unaffected side (P < 0.001). On both sides, P_WB_, P_SP_ extremely significantly increased (P < 0.001), whereas P_SW_, P_SL_, L_G_, L_S_, P_MAX-F_, P_MAX-A_, P_MAX-H_ extremely significantly decreased (P < 0.001) (Table 3) (Figure 4). Additionally, compared with the unaffected side, P_S_, P_WB_, P_SL_, P_SP_, L_G_, L_S_, P_MAX-F_, P_MAX-A_, P_MAX-H_ of the affected side decreased extremely significantly (P < 0.001), while P_SW_ increased extremely significantly (P < 0.001) (Table 4) (Figure 5).

### 3.2 Comparison of the ACLR group and the NC group at 2 months post-operation

**FIGURE 6:**
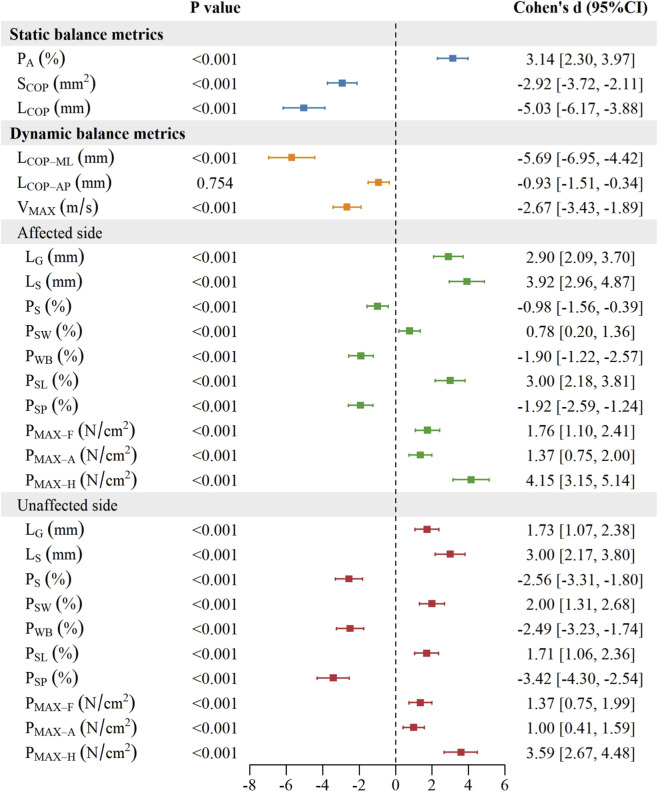
Comparison of balance parameters between the ACLR group and the NC group at 2 months post-operation. The forest plot shows the P-value, Cohen’s d, 95% CI of the balance parameters and direction indicates the ACLR group vs. the NC group.

**FIGURE 7:**
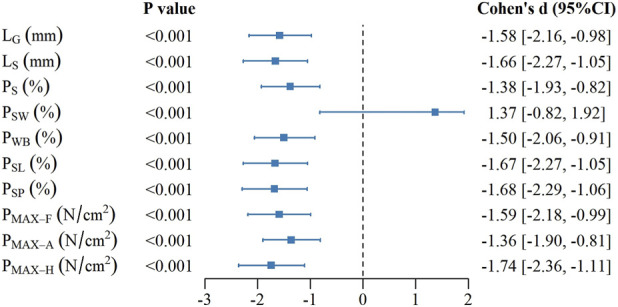
Comparison of balance parameters within the ACLR group at 2 months post-operation. The forest plot shows the P-value, Cohen’s d, 95% CI of the balance parameters and direction indicates affected side vs. unaffected side within the ACLR group.

Compared with the NC group, the ACLR group showed extremely significantly decreased P_A_ and increased S_COP_ and L_COP_ (P < 0.001) (Table 5). L_COP-ML_ and V_MAX_ of the ACLR group increased extremely significantly during walking (P < 0.001), while L_COP-AP_ showed no significant difference (P > 0.05). P_S_, P_WB_, P_SP_ on both sides of the ACLR group increased extremely significantly (P < 0.001) and P_SW_, P_SL_, L_G_, L_S_, P_MAX-F_, P_MAX-A_, P_MAX-H_ decreased extremely significantly (P < 0.001) (Table 6) (Figure 6). In addition, compared with the unaffected side, P_S_, P_WB_, P_SL_, P_SP_, L_G_, L_S_, P_MAX-F_, P_MAX-A_, P_MAX-H_ of the affected side decreased extremely significantly (P < 0.001), while P_SW_ increased extremely significantly (P < 0.001) (Table 7) (Figure 7).

### 3.3 Comparison of the ACLR group and the NC group at 6 months post-operation

**FIGURE 8:**
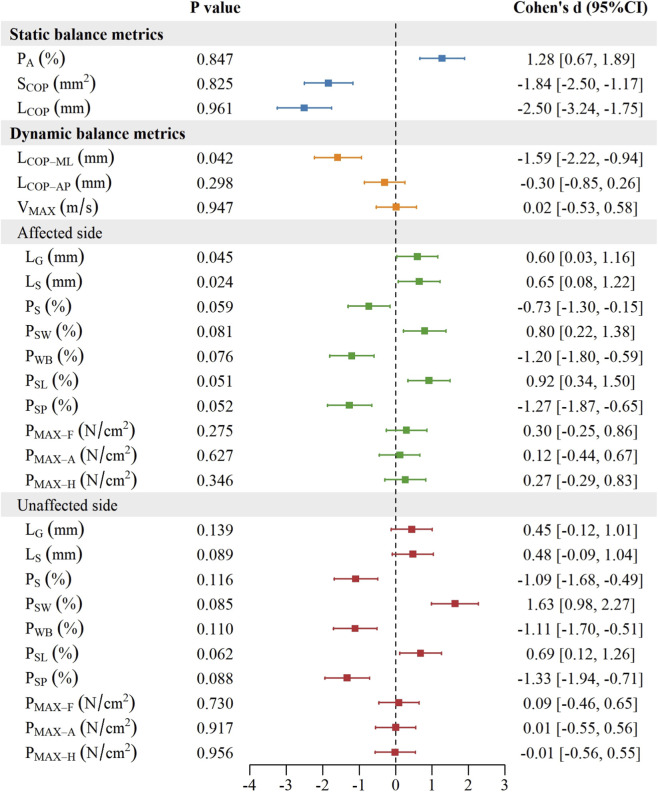
Comparison of balance parameters between the ACLR group and the NC group at 6 months post-operation. The forest plot shows the P-value, Cohen’s d, 95% CI of the balance parameters and direction indicates the ACLR group vs. the NC group.

**FIGURE 9:**
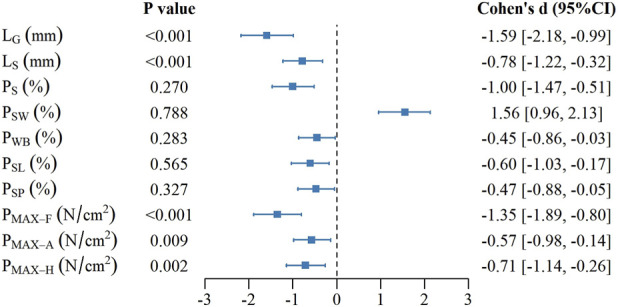
Comparison of balance parameters within the ACLR group at 6 months post-operation. The forest plot shows the P-value, Cohen’s d, 95% CI of the balance parameters and direction indicates affected side vs. unaffected side within the ACLR group.

During static standing, there were no statistically significant differences in P_A_, S_COP_ and L_COP_ between the ACLR group and the NC group (P > 0.05) (Table 8). During dynamic walking, the ACLR group showed a significant increase in L_COP-ML_ relative to the NC group (P < 0.05) (extremely large effect size, Cohen’s d > 1.2). V_MAX_ and L_COP-AP_ did not differ significantly between two groups (P > 0.05). On the affected side of the ACLR group, both L_G_ and L_S_ were significantly lower than those in the NC group (P < 0.05) [L_G_: medium effect size, Cohen’s d = 0.6, 95%CI (0.03, 1.16), L_S_: medium effect size, Cohen’s d = 0.65, 95%CI (0.08, 1.22)]. Similarly, L_G_ and L_S_ on the unaffected side of the ACLR group did not differ significantly from those of the NC group (P > 0.05). There were no statistically significant differences in bilateral P_S_, P_SW_, P_WB_, P_SL_, P_SP_, P_MAX-F_, P_MAX-A_, and P_MAX-H_ between the ACLR group and the NC group (P > 0.05) (Table 9) (Figure 8). Within the ACLR group, the affected side exhibited extremely significant decreases in L_G_, L_S_, and P_MAX-F_ compared with the unaffected side (P < 0.001) [L_G_: extremely large effect size, Cohen’s d = −1.59, 95%CI (−2.18, −0.99), L_S_: medium effect size, Cohen’s d = −0.78, 95%CI (−1.22, −0.32), P_MAX-F_: extremely large effect size, Cohen’s d = −1.35, 95%CI (−1.89, −0.80)]. P_MAX-A_ and P_MAX-H_ were significantly lower on the affected side as well (P < 0.05) [P_MAX-A_: medium effect size, Cohen’s d = −0.57, 95%CI (−0.98, −0.14), P_MAX-H_: medium effect size, Cohen’s d = −0.71, 95%CI (−1.14, −0.26)]. There were no statistically significant differences in P_S_, P_SW_, P_WB_, P_SL_, and P_SP_ (P > 0.05) (Table 10) (Figure 9).

## 4 Discussion

The current literature on preoperative and postoperative balance function assessment remains relatively limited. Most studies focus primarily on overall postural control, with fewer investigations incorporating detailed bilateral comparisons and comparisons with healthy populations (Lennon et al., 2025; Girdwood et al., 2025; Dai et al., 2021; Lehmann et al., 2021). This oversight hampers the detection of subtle functional defects. To address these gaps, our study employed the advanced SMARTX-MFS plantar pressure and gait assessment system to precisely quantify both dynamic and static balance indicators. This approach provided biomechanical reference data to optimize clinical rehabilitation protocols and refine functional assessments. Furthermore, by integrating our empirical findings with relevant literature, we provided an in-depth discussion of potential underlying mechanisms to comprehensively analyze these results. However, it must be emphasized that since our study did not include direct neurophysiological measurements, this interpretive framework served as a hypothesis-generating explanation rather than a directly demonstrated finding, aiming to provide a theoretical reference for phase-specific clinical interventions and the subsequent long-term observation of balance parameter changes.

### 4.1 Equilibrium characteristic analysis

#### 4.1.1 Analysis of preoperative equilibrium characteristics

During static standing, the ACLR group exhibited a significant decrease in P_A_ compared to the NC group. This finding indicated the ACLR group tendency to reduce the weight-bearing on the affected side during standing following an ACL injury (Chmielewski et al., 2002; Fernandes et al., 2016). Additionally, there were significant increases in S_COP_ and L_COP_, indicating that the COP was considerably unstable, thereby increasing the challenge of balance control (Soltani et al., 2014).

In the ACLR group, compared to the NC group, the gait cycles of both lower limbs exhibited increased P_S_ and decreased P_SW_. Following an ACL injury, the altered neuromuscular control, potentially involving a reduced capacity for eccentric quadriceps contraction, could contribute to a stiffer knee posture. Consequently, the prolonged P_S_ might serve as a functional adaptation to safely distribute joint loads and facilitate COP transfer during the gait cycles, which mathematically results in a proportional compression of the P_SW_ (Pietrosimone et al., 2022; Shelburne et al., 2005; Berchuck et al., 1990; Shelburne et al., 2004; Vakula et al., 2022). During the three phases of the P_S_, the ACLR group exhibited increased P_WB_, increased P_SP_ and decreased P_SL_ in bilateral lower limbs compared to the NC group. The prolonged P_WB_ might reflect a cautious, stiffer-limb gait pattern to safely absorb impact forces. Subsequently, the observed decrease in P_SL_ might be related to postural instability and elevated sensorimotor demands. This reduction indicated a strategy to minimize the duration of unilateral stance, potentially to manage perceived joint instability. Finally, the increased P_SP_ could provide the ACLR group with more time to safely and gradually transfer their body weight to the contralateral limb prior to initiating the P_SW_ (Pietrosimone et al., 2022; Shelburne et al., 2005; Berchuck et al., 1990; Shelburne et al., 2004; Vakula et al., 2022). Changes in the unaffected side are generally considered adaptive, aiming to preserve kinematic symmetry between the bilateral lower limbs (Ferber et al., 2004). Within the ACLR group, the affected side showed a decrease in P_S_, P_WB_, P_SL_, and P_SP_ compared to the unaffected side, whereas P_SW_ increased. The asymmetry in gait cycles of bilateral lower limbs might reflect a protective mechanism in the ACLR group to reduce joint loads on the affected side (Shelburne et al., 2005; Berchuck et al., 1990; Shelburne et al., 2004; Aghdam et al., 2022; Gokeler et al., 2012; Pap et al., 1999).

In the ACLR group, P_MAX-F_, P_MAX-A_, and P_MAX-H_ of bilateral lower limbs were decreased compared to the NC group, with the affected side exhibiting lower values than the unaffected side. The generalized reduction in bilateral plantar pressure might be related to the above protective gait strategy and altered foot roll-off mechanism, potentially aimed at diminishing vertical ground reaction forces to minimize joint impact (Pietrosimone et al., 2022; Huang et al., 2017; Çeti̇N et al., 2017; Berchuck et al., 1990; Urbach et al., 1999; Devita et al., 1997; Torry et al., 2000). Similarly, the protective gait strategy, potentially associated with alterations in neuromuscular control, might contribute to the significant bilateral shortening of the L_G_ and L_S_ observed in the ACLR group, particularly on the affected side (Park et al., 2021; Knoll et al., 2004; Sharifi et al., 2024; Sugawara et al., 2016; Lisee et al., 2025; Moraiti et al., 2007).

The observation of a higher V_MAX_ suggested a rapid transition of the COP during walking, potentially serving as a compensatory strategy to enhance gait stability (Huang et al., 2017; Çeti̇N et al., 2017; Berchuck et al., 1990). The notable increase in L_COP-AP_ might stem from the compromised ability of the ACL to constrain tibial advancement post-injury. Consequently, the ACLR group might subconsciously limit tibial advancement by leaning forward or flexing at the hips. Furthermore, the elevated L_COP-ML_ might be associated with compensatory pelvic lateral tilt during walking. As suggested by existing studies, suboptimal coordination of the proximal kinetic chain during walking might contribute to the observed increase in medial-lateral displacement of the COP (Palmieri-Smith et al., 2009; Granach et al., 2013).

#### 4.1.2 Analysis of 2 months postoperative balance characteristics

Two months postoperatively, the ACLR group exhibited a larger L_COP_ and S_COP_, alongside a lower P_A_ compared to the NC group. Given that neural reinnervation within the graft is typically incomplete at this early stage, the observed asymmetric weight distribution and increased COP sway might relate to a residual deficit in precise somatosensory feedback (Soltani et al., 2014; Attalin et al., 2024; Parus et al., 2015; Akbari et al., 2015).

The ACLR group exhibited a significant increase in the P_S_, P_WB_, and P_SP_, alongside a significant decrease in the P_SW_ and P_SL_ across bilateral lower limbs compared to the NC group. Within the ACLR group, the affected side exhibited significantly shorter durations in the P_S_, P_WB_, P_SL_ and P_SP_, while spending proportionally more time in the P_SW_ compared to the unaffected side. This persistent compensatory gait pattern closely resembled the preoperative state, which might reflect an ongoing alteration in whole-body neuromuscular control to prioritize dynamic stability (Pietrosimone et al., 2022; Aghdam et al., 2022; Gokeler et al., 2012; Pap et al., 1999; Schliemann et al., 2018; Lin et al., 2025; Forelli et al., 2025). In addition, these spatiotemporal gait adaptations at this stage might also be related to psychological factors such as kinesiophobia (Lisee et al., 2025).

The ACLR group exhibited shortened L_G_ and L_S_, alongside decreased P_MAX-F_, P_MAX-A_, and P_MAX-H_ across bilateral lower limbs compared to the NC group, with the affected side exhibiting lower values than the unaffected side. These changes were closely related to the protective gait strategies during the early postoperative stage. Specifically, the shortened L_G_ might indicate an altered foot roll-off mechanism, potentially associated with persistent knee joint stiffness to protect the healing graft from abnormal shear forces (Matov et al., 2025; Shelburne et al., 2005; Lepley AS. et al., 2015; Garcia et al., 2022; Pfeiffer et al., 2018). Similarly, the shortened L_S_ suggested an adaptation to minimize the duration of single-limb support, which could serve to manage perceived knee joint instability (Shelburne et al., 2005; Sugawara et al., 2016). Concurrently, the decrease in P_MAX-H_ reflected a softer initial contact strategy, which could be related to potential quadriceps weakness (Pietrosimone et al., 2022; Vakula et al., 2022). Furthermore, the decrease in P_MAX-F_ suggested a reduction in lower-limb propulsive efficiency during terminal stance (Huang et al., 2017; Sharifi et al., 2024). Moreover, the reduction in P_MAX-A_, a parameter associated with arch support and shock absorption, potentially reflected a kinetic adaptation intended to reduce the upward transmission of vertical ground reaction forces, thereby mitigating excessive joint loading (Bowersock et al., 2017; Capin et al., 2018; Bjornsen et al., 2025). Finally, the unaffected side adaptively matched the restricted kinematic pattern of the affected side, potentially driven by alterations in neuromuscular control (Bowersock et al., 2017; Johnson et al., 2023).

Notably, compared to the NC group, there was no significant statistical difference in the L_COP-AP_, which suggested that the ACLR might have successfully provided immediate mechanical restraint against anterior tibial translation in the sagittal plane (Kösters et al., 2020). However, the increased L_COP-ML_ and V_MAX_ persisted in the ACLR group. The persistence of these abnormalities could indicate that compensatory kinematic adaptations—such as the altered hip strategies and protective COP shifts documented in the preoperative stage—might remain unresolved in the early postoperative stage (Wang et al., 2023; Palmieri-Smith et al., 2009; Granach et al., 2013; Kösters et al., 2020; Lehmann et al., 2017; Vaz et al., 2023; Shi et al., 2015).

#### 4.1.3 Analysis of 6 months postoperative balance characteristics

Six months postoperatively, no significant differences were observed in static balance indicators (P_A_, L_COP_, S_COP_) between the ACLR group and the NC group. This recovery might be primarily driven by central neuroplastic adaptations, wherein an increased reliance on alternative sensory inputs, such as vision and foot tactile feedback, might help compensate for the permanent loss of native ligament mechanoreceptors (Needle et al., 2017; Hallaj Mazidluie et al., 2024). However, while achieving static equilibrium is a positive early rehabilitation milestone, sole reliance on these static tests may provide an incomplete clinical assessment. Normal static parameters do not inherently guarantee that the knee possesses the reflexive dynamic stability required for athletic movements, potentially masking residual dynamic vulnerabilities (Brophy et al., 2022; Akbari et al., 2015).

Regarding dynamic balance indicators, the ACLR group showed no statistically significant differences in the bilateral gait cycles, maximum plantar pressures, L_COP-AP_, and V_MAX_ compared to the NC group. Within the ACLR group, there were no significant differences in the bilateral gait cycles. This recovery might reflect an initial recalibration of locomotor patterns, potentially driven by the reinforcement of daily walking and the restoration of mechanical stability (Guertin, 2013; Di Russo et al., 2023; Grooms et al., 2017; Rodriguez et al., 2021; Scarano et al., 2026; Schnit et al., 2025; Ellis et al., 2013; Southall et al., 2026). Notably, L_COP-ML_ remained elevated in the ACLR group, suggesting a residual deficit in frontal-plane postural control. In addition, although there were no statistically significant differences in P_MAX-F_, P_MAX-A_, and P_MAX-H_ of the bilateral lower limbs between the ACLR group and the NC group, intra-group comparisons revealed that these values—especially the P_MAX-H_—remained lower on the affected side compared to the unaffected side. Furthermore, compared with the NC group, the L_G_ and L_S_ of the affected side in the ACLR group decreased, while no significant difference was observed in the unaffected side. Within the ACLR group, L_G_ and L_S_ remained significantly shorter on the affected side compared to the unaffected side.

Overall, these findings indicated that deficits in dynamic balance persisted. Mechanistically, the asymmetry between static and dynamic balance recovery might be associated with the central-peripheral loop plasticity and the subsequent adoption of compensatory muscle recruitment strategies (Scarano et al., 2026; Wei et al., 2026; Lee et al., 2026). During highly predictable, low-demand tasks such as static standing, these putative cortically-driven compensatory strategies might effectively maintain equilibrium, thereby masking underlying functional deficits (Moiroux-Sahraoui et al., 2025; Wei et al., 2026). However, during the complex, multi-planar biomechanical demands of dynamic walking, such hypothesized neural adaptations might not fully replicate fluid, reflexive motor control (Scarano et al., 2026; Schnit et al., 2025). Clinically, these persistent dynamic asymmetries warranted careful consideration. As proposed in current literature, the enduring pattern of kinematic and kinetic underloading could potentially alter articular cartilage metabolism, serving as a contributing biomechanical factor to the accelerated onset of PTOA (Teng et al., 2017; Pietrosimone et al., 2017; Pfeiffer et al., 2019).

### 4.2 Analysis of rehabilitation guidance

The longitudinal assessment of balance characteristics via high-resolution plantar pressure analysis revealed stage-specific biomechanical deficits throughout the ACLR recovery process. Recognizing and addressing these deficits is paramount for optimizing evidence-based clinical rehabilitation protocols.

Preoperatively: Our findings demonstrated substantial static and dynamic balance impairments following an ACL injury. Given these pervasive deficits, the preoperative phase should be regarded as a critical period for targeted rehabilitation rather than a passive waiting phase. Extensive literature demonstrates the efficacy of robust prehabilitation—integrating targeted neuromuscular control exercises and progressive quadriceps strengthening—to address the specific compensatory adaptations (Carter et al., 2020; Giesche et al., 2020; Culvenor et al., 2022; Failla et al., 2016). For instance, real-time visual biofeedback during weight-bearing tasks could be employed to directly target the observed static instability and the tendency to unload the affected side, thereby promoting bilateral load symmetry (Kal et al., 2023; Marshall et al., 2020). Furthermore, to address kinetic alterations—such as the blunted plantar pressures and compensatory gait cycles—neuromuscular electrical stimulation (NMES) may be beneficial. NMES is often utilized in current practice to mitigate potential quadriceps weakness by directly activating motor neurons circumventing spinal and cortical inhibition (Culvenor et al., 2022; Arhos et al., 2024; Toth et al., 2020). Additionally, targeted proprioceptive interventions, such as threshold to detection of passive motion (TTDPM) and perturbation training, could be implemented to challenge the rigid, protective knee postures identified during walking. Such interventions may help the ACLR group manage perceived joint instability and normalize altered COP trajectories (Kim et al., 2017; Lee et al., 2009; Hartigan et al., 2009; Chmielewski et al., 2005).

Two months postoperatively: Although our data suggested that ACLR effectively restored mechanical sagittal-plane stability at this early rehabilitation juncture, significant balance deficits persisted. If left unaddressed, these compensatory motor patterns may persist, potentially hindering optimal functional recovery and delaying safe progression to subsequent training phases and return to sport (RTS) (Moiroux-Sahraoui et al., 2025; Southall et al., 2026). To address the observed compensatory spatiotemporal gait adaptations—potentially associated with ongoing quadriceps weakness—interventions such as applying high-frequency NMES during functional closed-kinetic-chain movements and low-load blood flow restriction (BFR) training may be beneficial. Current literature indicates that these modalities can help mitigate muscle atrophy without jeopardizing graft integrity, thereby facilitating the restoration of more symmetrical load acceptance and push-off forces (Kim et al., 2010; Labanca et al., 2022; Labanca et al., 2018; Lepley LK. et al., 2015; Hauger et al., 2018; Li et al., 2025; Wilk et al., 2024; Kong et al., 2022; Ortega-Prados et al., 2025; Buckthorpe et al., 2024). Additionally, integrating real-time visual biofeedback during treadmill retraining could be employed to target the persistent asymmetric plantar pressure, directing attention to external cues to promote bilateral symmetry (Wilk et al., 2024; Wellsandt et al., 2025; Molka et al., 2015). Furthermore, to manage the residual frontal-plane instability indicated by the elevated L_COP-ML_, rehabilitation should target the proximal kinetic chain, particularly the hip musculature. Integrating multi-planar, dual-task perturbation exercises may effectively challenge these rigid protective strategies, helping to normalize altered COP trajectories (Moiroux-Sahraoui et al., 2025; Burnham et al., 2026; Tarchichi et al., 2025; Irawan et al., 2026). Implementing such a targeted, data-driven optimization framework is anticipated to help correct these maladaptive motor patterns, establishing the biomechanical foundation required for subsequent advanced plyometric training and safe RTS (Matov et al., 2025; Southall et al., 2026; Naczk et al., 2026; Interlandi et al., 2026; Nagelli et al., 2020).

Six months postoperatively: Static balance metrics reached levels that were statistically indistinguishable from the NC group baselines. As noted previously, this recovery is often hypothesized in the literature to be supported by sensory reweighting and central neuroplasticity (Needle et al., 2017; Hallaj Mazidluie et al., 2024). However, interpreting this early static recovery as full functional restoration may provide an incomplete clinical assessment (Brophy et al., 2022; Akbari et al., 2015). Despite the ACLR group appearing statically stable, our intra-group analysis revealed that specific dynamic balance parameters remained significantly asymmetric. Current literature indicates that fewer than 15% of patients pass comprehensive RTS test batteries at six or even 9 months, primarily due to persistent quadriceps weakness and altered dynamic postural control during high-impact tasks (Welling et al., 2018; Toole et al., 2017; Webster and Feller, 2019). Consequently, clearing the ACLR group for RTS based purely on standard postoperative timeframes or static equilibrium may prematurely expose them to reinjury risks. Our findings reinforced the contemporary clinical consensus: to ensure safe progression to unrestricted RTS, clinicians should not rely solely on static evaluations. Instead, rehabilitation must integrate comprehensive dynamic biomechanical assessments that can expose residual multi-planar vulnerabilities, ensuring a more objective and robust functional recovery (Grindem et al., 2016; Beischer et al., 2020).

### 4.3 Limitations and future directions

This study has several limitations that merit careful consideration. First, the longitudinal follow-up was limited to 6 months after ACLR. Prior work shows that functional recovery after ACLR—especially full restoration of dynamic balance, multiplanar postural stability, and refined neuromuscular control—often continues well beyond 6–12 months, and RTS assessments are commonly performed at 9–12 months (Brophy et al., 2022; Kyritsis et al., 2016; Angelozzi et al., 2012). Therefore, although our 6-month findings illuminated the asymmetry between static and dynamic balance recovery, they did not reflect the long-term trajectory of these dynamic processes. Additionally, while the SMARTX-MFS plantar pressure system detected subclinical gait and plantar pressure asymmetries, this study lacked integration with simultaneous 3D kinematic tracking and surface electromyography. Integrating these modalities in future investigations is essential to translate our current hypothesis-generating interpretations into directly measured neurophysiological evidence.

Second, regarding statistical interpretation, this study was framed as an exploratory data analysis. To preserve the statistical sensitivity required for detecting residual subclinical deficits, adjustments for multiple comparisons (e.g., Bonferroni correction) were not applied. While the balance deficits observed early in the rehabilitation process were highly robust (mostly P < 0.001) and would likely survive such corrections, the unadjusted family-wise error rate inherently inflated the risk of Type I errors. Consequently, readers should interpret the statistical results with caution based on the Cohen’2s d effect sizes and 95% CIs, especially for variables exhibiting only marginally significant differences (P < 0.05) at the 6-month postoperative mark (e.g., L_COP-ML_, L_G_, L_S_, P_MAX-A_ and P_MAX-H_). Additionally, although our *post hoc* power analysis indicated that the sample size (n = 25 per group) was sufficient to detect substantial biomechanical deficits, the absence of an *a priori* sample size calculation based on the minimal clinically important difference remained a limitation. This omission increased the risk of type II errors, potentially limiting the statistical power to identify more subtle differences. Therefore, future large-scale confirmatory cohort studies are warranted to build upon the data from this exploratory research.

Third, we did not systematically account for limb dominance. We designated the surgical side as the “affected side” and the contralateral healthy leg as the “unaffected side”, but participants were not sub-classified based on dominant versus non-dominant status. Although current research indicates that minor asymmetry in the lower limbs is prevalent among the general population, the extent to which the dominant limb contributes to this phenomenon remains uncertain (D’Emanuele et al., 2025; Dos’Santos et al., 2019; Molitor et al., 2024; Polk et al., 2017; Brown et al., 2014). While the profound asymmetries observed preoperatively and 2 months postoperatively in our cohort vastly exceeded normal physiological variance and were indicative of pathological compensation, natural limb dominance might confound the interpretation of the subtle residual asymmetries observed at 6 months.

Fourth, the mean interval from ACL injury to ACLR in our ACLR group was relatively short (53.92 ± 19.95 days). The literature presents varying perspectives on how chronicity (greater than 3 months) affects proprioception: while some evidence suggests that chronic ACL injury leads to more pronounced postural stability deficits due to progressive mechanoreceptor degeneration, other studies have found no significant correlation between injury duration and mechanoreceptor viability (Lee et al., 2015; Denti et al., 1994; Adachi et al., 2002; Sha et al., 2017). Because this longitudinal cohort primarily consisted of acute to subacute cases, the effects of varying chronicity on balance recovery could not be directly compared.

Fifth, although walking velocity was neither matched between groups nor statistically controlled as a covariate, several dynamic parameters are potentially speed-dependent. Decoupling primary neuromuscular deficits from secondary adaptations induced by altered walking velocity remains a significant challenge. However, existing biomechanical literature suggests that post-ACLR gait alterations often reflect genuine neuromuscular control deficits rather than mere speed-dependent artifacts (Garcia et al., 2022; Buck et al., 2024; Almansouri and Sigward, 2025; Knobel et al., 2021; Brebels et al., 2026). For instance, acutely normalizing walking speed to match the NC group (e.g., 1.3 m/s) fails to resolve characteristic joint kinetic asymmetries or quadriceps underloading (Buck et al., 2024; Almansouri and Sigward, 2025). Furthermore, increasing walking velocity has been shown to exacerbate vertical and posterior ground reaction force asymmetries in the ACLR group, whereas the NC group maintains symmetrical loading across velocity constraints (Garcia et al., 2022; Knobel et al., 2021; Brebels et al., 2026). This differential response suggests that our findings likely reflect underlying neuromuscular control deficits and protective movement adaptations. To systematically isolate these confounding factors, future studies should implement a multi-condition, speed-constrained design. Evaluating both the ACLR and the NC groups across self-selected and constrained fixed-velocity conditions (e.g., on a synchronized treadmill at preferred, +25%, and +50% velocity increments) will help establish speed-dependent thresholds and clarify their modulating effects on dynamic balance parameters.

Given these limitations, we intend to design a multi-modal, long-term cohort study building upon the current exploratory data. Future research will integrate Artificial Intelligence (AI)-driven biomechanical modeling and surface electromyography to evaluate the long-term trajectories and underlying mechanisms of dynamic asymmetries at 12 and 24 months. Additionally, we will include limb dominance, the injury-to-surgery interval, and walking velocity as covariates in our analysis to better assess these variables in relation to RTS readiness and long-term knee joint health.

## 5 Conclusion

Preoperatively, the ACLR group exhibited pronounced deficits in both static and dynamic balance. Two months postoperatively, only the L_COP-AP_ showed no statistically significant difference compared with the NC group, suggesting restored sagittal mechanical stability, while other balance deficits persisted. Six months postoperatively, the statistically significant differences in static balance indicators between the two groups had resolved, but specific dynamic balance indicators remained abnormal. Inter-group comparisons revealed that the ACLR group continued to exhibit increased L_COP-ML_ alongside decreased L_G_ and L_S_ on the affected side. Intra-group comparisons demonstrated concurrent reductions in L_G_, L_S_, P_MAX-F_, P_MAX-A_ and P_MAX-H_ on the affected side. In the context of clinical rehabilitation, these objective biomechanical assessment indicators provided quantitative benchmarks for identifying stage-specific balance deficits. Future research should integrate multimodal three-dimensional kinematics to elucidate the exact motor-control mechanisms underlying these persistent deficits, thereby refining evidence-based rehabilitation criteria.

## Data Availability

The original contributions presented in the study are included in the article/Supplementary Material, further inquiries can be directed to the corresponding authors.
