# Supplementary material for: Evolution of pre- and post-operative balance characteristics in patients undergoing anterior cruciate ligament reconstruction: implications for rehabilitation
Source: Front Bioeng Biotechnol. 2026 Jun 25;14:1861366. doi: 10.3389/fbioe.2026.1861366 (PMC13346059; doi:10.3389/fbioe.2026.1861366)
Supplement: Supplementary file 1 [file Table1.pdf]

Supplementary Table S1. The ICC values and 95% CIs of the measurement results from a single test conducted by three operators on the same 25 participants respectively

	ICC	95% CI
P_A (%)	0.922	(0.857, 0.962)
L_{COP} (mm)	0.913	(0.840, 0.957)
S_{COP} (mm^2)	0.884	(0.790, 0.943)
L_{COP-AP} (mm)	0.909	(0.825, 0.957)
L_{COP-ML} (mm)	0.894	(0.808, 0.948)
V_{MAX} (m/s)	0.893	(0.805, 0.947)
Right - P_S (%)	0.879	(0.783, 0.940)
Left - P_S (%)	0.913	(0.840, 0.958)
Right - P_{SW} (%)	0.862	(0.748, 0.932)
Left - P_{SW} (%)	0.904	(0.824, 0.953)
Right - P_{WB} (%)	0.906	(0.829, 0.954)
Left - P_{WB} (%)	0.800	(0.656, 0.898)
Right - P_{SL} (%)	0.892	(0.805, 0.947)
Left - P_{SL} (%)	0.855	(0.742, 0.927)
Right - P_{SP} (%)	0.854	(0.742, 0.927)
Left - P_{SP} (%)	0.865	(0.758, 0.933)
Right - L_G (mm)	0.910	(0.834, 0.956)
Left - L_G (mm)	0.892	(0.803, 0.947)
Right - L_S (mm)	0.895	(0.809, 0.948)
Left - L_S (mm)	0.901	(0.820, 0.951)
Right - P_{MAX-F} (N/cm^2)	0.919	(0.851, 0.960)
Left - P_{MAX-F} (N/cm^2)	0.911	(0.836, 0.956)
Right - P_{MAX-A} (N/cm^2)	0.858	(0.747, 0.929)
Left - P_{MAX-A} (N/cm^2)	0.840	(0.719, 0.920)
Right - P_{MAX-H} (N/cm^2)	0.903	(0.822, 0.952)
Left - P_{MAX-H} (N/cm^2)	0.883	(0.788, 0.942)

Supplementary Table S2. The ICC values and 95% CIs of the measurement results of two tests (with an interval of one day) on the same 25 participants conducted by the same operator

	ICC	95% CI
P_A (%)	0.947	(0.884, 0.976)
L_{COP} (mm)	0.922	(0.832, 0.965)
S_{COP} (mm^2)	0.872	(0.732, 0.941)
L_{COP-AP} (mm)	0.922	(0.831, 0.965)
L_{COP-ML} (mm)	0.861	(0.710, 0.936)
V_{MAX} (m/s)	0.919	(0.825, 0.963)
Right - P_S (%)	0.953	(0.896, 0.979)
Left - P_S (%)	0.900	(0.789, 0.954)

Right - P_{SW} (%)	0.939	(0.868, 0.972)
Left - P_{SW} (%)	0.851	(0.690, 0.931)
Right - P_{WB} (%)	0.894	(0.774, 0.952)
Left - P_{WB} (%)	0.819	(0.632, 0.916)
Right - P_{SL} (%)	0.875	(0.737, 0.943)
Left - P_{SL} (%)	0.834	(0.660, 0.923)
Right - P_{SP} (%)	0.839	(0.670, 0.925)
Left - P_{SP} (%)	0.875	(0.737, 0.943)
Right - L_G (mm)	0.931	(0.852, 0.969)
Left - L_G (mm)	0.948	(0.886, 0.977)
Right - L_S (mm)	0.950	(0.891, 0.978)
Left - L_S (mm)	0.940	(0.868, 0.973)
Right - P_{MAX-F} (N/cm^2)	0.923	(0.833, 0.965)
Left - P_{MAX-F} (N/cm^2)	0.915	(0.817, 0.962)
Right - P_{MAX-A} (N/cm^2)	0.816	(0.626, 0.915)
Left - P_{MAX-A} (N/cm^2)	0.798	(0.596, 0.906)
Right - P_{MAX-H} (N/cm^2)	0.957	(0.905, 0.981)
Left - P_{MAX-H} (N/cm^2)	0.920	(0.828, 0.964)
